# Single-incision thoracoscopic surgery for primary spontaneous pneumothorax

**DOI:** 10.1186/1749-8090-6-58

**Published:** 2011-04-21

**Authors:** Pin-Ru Chen, Chien-Kuang Chen, Yu-Sen Lin, Hsu-Chih Huang, Jian-Shun Tsai, Chih-Yi Chen, Hsin-Yuan Fang

**Affiliations:** 1 Division of Thoracic Surgery, Department of Surgery, China Medical University Hospital, China Medical University, Taichung, Taiwan

## Abstract

**Objective:**

Single-incision laparoscopic surgery had been proven effective for appendectomy, cholecystectomy, and inguinal hernia repair. However, single-incision thoracoscopic surgery (SITS) in primary spontaneous pneumothorax (PSP) has not been reported.

**Methods:**

We prospectively enrolled 30 PSP patients who received thoracoscopic surgery in the division of Thoracic Surgery of China Medical University Hospital. Ten patients received SITS and 20 patients received traditional three-port thoracoscopic surgery. The operative time, blood loss, wound size, visual analog scale (VAS) pain score, and patient satisfaction score were compared.

**Results:**

There was no significant difference in the operative time and blood loss between the two groups. However, the VAS pain scores were significantly better in the SITS group in first 24 hours after surgery. Patient satisfaction scores in the SITS group were also significantly better in the first 24 and 48 hours after operation.

**Conclusion:**

Although three-port thoracoscopic surgery for PSP is well established, SITS results in better patient satisfaction and decreased postoperative pain in the treatment of PSP.

## Introduction

Primary spontaneous pneumothorax (PSP) is a perplexing disease that usually occurs in young, otherwise healthy individuals without clinically apparent lung disease in their late teens or third decade of life [1]. It is defined by the presence of air in the pleural cavity with secondary lung collapse, and occurs without a preceding event or obvious precipitating cause [2]. The incidence of PSP is approximately 7 to 18 and 1 to 6 cases per 100,000 individuals per year in males and females, respectively [3]. Surgical resection of the blebs or bullae could decrease the recurrent rate.

Thoracoscopic surgical techniques have transformed may surgical procedures over recent decades. Minimal access techniques allow extensive operations to be performed with little trauma, leading to faster recovery times and shorter hospital stays [4]. In abdominal surgery, using of single-incision laparoscopic surgery (SILS) for appendectomy, cholecystectomy, and inguinal hernia repaired have been reported [5-7]. However, single-incision thoracoscopic surgery (SITS) has not been reported. We herein describe our technique for performing SITS in patients with PSP and compare outcomes with three-port video-assist thoracoscopic surgery (VATS).

## Patients and methods

Between March 2009 and July 2009, we prospectively enrolled 30 consecutive PSP patients who received thoracoscopic surgery in the division of Thoracic Surgery of China Medical University Hospital in central Taiwan. PSP was defined as spontaneous air accumulation in the pleural cavity without evidence of clinical lung disease. The surgical indications were 1) recurrent episode, 2) persist air leakage for more than 4 -5 days, and/or 3) abnormal radiographic findings. The inclusion criteria of patients were 1) pneumothorax noted by chest radiography or chest computerized tomography (CT) scan on admission to the hospital, 2) patient was between 15 and 40 years of age, 3) no history of lung diseases, such as chronic obstructive pulmonary disease, asthma, pulmonary fibrosis, or pneumoconiosis, and 4) no history of other systemic diseases, such as uremia, liver cirrhosis, malignancy, or chronic heart and liver diseases. The exclusion criteria were 1) history of chest trauma, such as rib fracture and pulmonary contusion, 2) history of pneumonia or pulmonary tuberculosis, and 3) history of pulmonary surgery, including lobectomy, segmentectomy, and wedge resection of the lung. PSP was treated by a wedge resection of the lung using VATS or SITS. Each patient was fully informed about the difference of these two methods, such as single incision and three incisions. The other procedures between these two methods are all the same. Surgical risks, potential complications were also informed. Written informed consents were obtained. The study was approved by the Institutional Review Board of the China Medical University. All patients were followed for at least 3 months postoperatively in the outpatient department.

### Surgical technique

#### SITS

**Figure 1:**
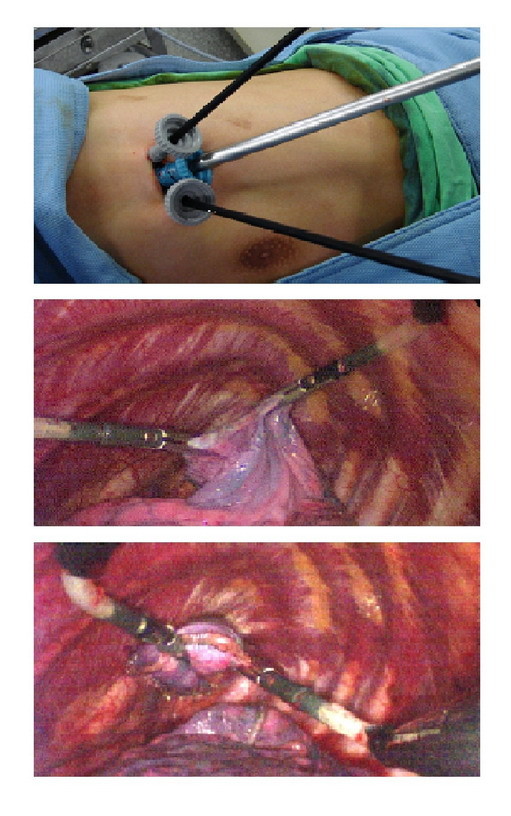
**Surgical approach for primary spontaneous pneumothorax (PSP) in single incision thoracoscopic surgery (SITS)**.

SITS was performed with the patient under general anesthesia using one-lung ventilation. The patient was placed in a lateral position. A skin incision was made 2.5 cm in length through the previous chest thoracostomy wound (4th, 5th, or 6th intercostal space) for insertion of a video-thoracoscope through an 11-mm thoracoport. With the lung deflated, the other two 5-mm thoracoports were inserted next to the 11-mm thoracoport (Figure 1). The visceral blebs and bullae were excised using a Endo GIA 60 stapler (Autosuture, United States Surgical Corporation). The subsequent mechanical pleurodesis was performed with a scouring pad on the tip of a forceps. After checking for air leaks and bleeding, one pig-tail drainage tube was inserted through the incision and connected to an underwater sealing drain with a suction of 15 cm H_2_O.

#### VATS

VATS was performed with the patient was under general anesthesia using one-lung ventilation. The patient was placed in a lateral position. Three small incisions were used. An initial skin incision was made 1.5 cm in length through the previous chest thoracostomy wound (5th or 6th intercostal space) for video-thoracoscope insertion. With the lung deflated, the other two incisions, 0.5 cm in length, were made along the anterior-axillary line (4th or 5th intercostal space) and the mid-axillary line (3rd or 4th intercostal space). The visceral blebs and bullae were excised using an Endo GIA 60 stapler (Autosuture). The subsequent mechanical pleurodesis was performed with a scouring pad on the tip of forceps. After checking for air leaks and bleeding, one pig-tail drainage tube was inserted through the incision and connected to an underwater sealing drain with a suction of 15 cm H_2_O.

### Visual analog scale (VAS) score

The intensity of postoperative pain was determined using a VAS score [8]. The VAS scale was an unlabeled 10-cm horizontal line with word anchors at each end, ranging from 0 = "no pain at all" to 10 = "pain as bad as it could be." The patients were asked to make a mark on the line representing the maximum pain intensity experienced since the last scoring. This mark was converted to distance in centimeters from the "no pain" anchor to give a pain score that could range from 0 to 10 cm. Pain scores were taken 24, 48, and 72 h after surgery. As the primary outcome variable, we calculated the mean pain score at each of these 3 times.

### Patient satisfaction scale

All the patients were given a form showing 4 grades (excellent = 1, good = 2, fair = 3, and poor = 4) and they were asked to freely evaluate the clinical outcome. Patient satisfaction scores were taken 24, 48, 72 h, and one month after surgery. Postoperative patient satisfaction data were collected by an independent team that did not take part in the operative procedures.

### Statistical analysis

Categorical variables were expressed as percentages and continuous variables were expressed as medians ± standard deviation. Continuous variables were compared by Mann-Whitney U test and categorical variables were compared by chi-square test or the Fisher's exact test (when the expected number of an analysis cell was smaller than or equal to 5). Statistical analysis was performed by using SPSS software (version 12.0, SPSS Inc., Chicago, Illinois, USA). Statistical significance was set at *p *< 0.05.

## Results

### Patient characteristics

Ten patients received SITS and 20 patients received three-port VATS. The mean age of the PSP patients was 22.97 ± 8.13 years (range, 15 to 40 years), and there were 28 men and 2 women. Demographic data were shown in Table 1. Eight patients (27%) were smokers, of whom the smoking duration and cigarette consumption were 5.5 ± 2.5 years and 1.2 ± 0.3 packages per day, respectively. There were no significant differences between the SITS and VATS groups.

**Table 1:** Clinical characteristics of primary spontaneous pneumothorax (PSP) patients in single incision thoracoscopic surgery (SITS) and video-assisted thoracoscopic surgery (VATS)

	SITS Group (n = 10)	VATS Group (n = 20)	P-value
Age (years)	20.50 ± 5.54	24 ± 9.02	0.246
Gender			
Male	9 (90%)	19(95%)	0.605
Female	1 (10%)	1(5%)	
Weight (kg)	59.47 ± 10.35	57.68 ± 4.78	0.518
Height (cm)	173.77 ± 8.82	172.34 ± 6.21	0.609
Side involved			
Right	3 (30%)	6 (30%)	1.000
Left	7 (70%)	14 (70%)	
Bilateral	0 (0%)	0 (0%)	
Smoking			
No	9 (90%)	13 (65%)	0.144
Yes	1 (10%)	7 (35%)	

Values are medians ± standard deviation for continuous variables or # cases (%) for categorical variables. P-values from Mann-Whitney U test (continuous variables), Chi-square test (categorical variables) or the Fisher's exact test.

Surgical characteristics of PSP are presented in Table 2. Surgical indications for PSP were ipsilateral recurrence (77%), persistent air leakage (10%), and contralateral recurrence (13%). The mean surgical time was 83 ± 21 min, and mean postoperative hospital stay was 4.6 ± 1.2 days. No deaths occurred, and no full thoracotomy was needed during or after surgery. Two patients (6.7%) had air leaks, and were managed conservatively. There were 28 patients who had some blebs at the apex, including 2 patients who had some blebs at the lower lobe. Two patients had no blebs at the apex of the lung. Microscopically, subpleural blebs were found in 27 (90%) specimens. There were no significant differences in operative time, blood loss, postoperative drainage, and postoperative hospital stay between the two groups. There were no recurrences during the follow-up period.

**Table 2:** Surgical characteristics of primary spontaneous pneumothorax (PSP) patients in single incision thoracoscopic surgery (SITS) and video-assisted thoracoscopic surgery (VATS)

	SITS Group (n = 10	VATS Group (n = 20)	P-value
Surgical indications			
Ipsilateral recurrence	7 (70%)	16 (80%)	
Persistent air leaks	2 (20%)	1 (5%)	
Contralateral recurrence	1 (10%)	3 (15%)	0.425
Hemopneumothorax	0 (0%)	0 (0%)	
Other	0 (0%)	0 (0%)	
Presence of bleb			
Yes	9 (90%)	19 (95%)	0.605
No	1 (10%)	1 (5%)	
Operation time (min)	80.50 ± 20.74	85.50 ± 21.87	0.553
Length of stay (days)	7.80 ± 2.74	7.90 ± 2.22	0.915
Post operation hospital stay (days)	4.40 ± 0.96	4.85 ± 1.46	0.387
Blood loss (mL)	minimal	minimal	
Post operative drainage (days)	3.00 ± 0.94	3.55 ± 1.50	0.301

Values are medians ± standard deviation for continuous variables or # cases (%) for categorical variables. P-values from Mann-Whitney U test (continuous variables), Chi-square test (categorical variables) or the Fisher's exact test.

### VAS score

In the SITS group, the VAS scores at 24, 48, and 72 hours postoperatively were 4.50 ± 0.70, 4.20 ± 0.78, and 3.30 ± 0.60, respectively, whereas in the VATS group, the VAS scores were 4.95 ± 0.39, 4.25 ± 0.58, and 3.55 ± 0.60. The VAS score at 24 h was significantly different between the two groups (*p *= 0.032; Table 3).

**Table 3:** Visual analog scale (VAS) score and patient satisfactory scale of primary spontaneous pneumothorax (PSP) patients in single incision thoracoscopic surgery (SITS) and video-assisted thoracoscopic surgery (VATS)

	SITS Group (n = 10	VATS Group (n = 20)	P-value
Visual analog scale (VAS) score			
Preoperation			
24 hours	4.50 ± 0.70	4.95 ± 0.39	0.032*
48 hours	4.20 ± 0.78	4.25 ± 0.58	0.088
72 hours	3.30 ± 0.48	3.55 ± 0.60	0.265
Patient satisfactory scale			
24 hours	1.90 ± 0.74	2.55 ± 0.82	0.045*
48 hours	2.40 ± 0.52	2.90 ± 0.64	0.041*
72 hours	2.30 ± 0.94	2.45 ± 0.82	0.659

Values are medians ± standard deviation for variables. P-values from Mann-Whitney U test.

### Patient satisfaction scale

In the SITS group, the patient satisfaction scale scores at 24, 48, and 72 hours postoperatively were 1.90 ± 0.74, 2.40 ± 0.52, and 2.30 ± 0.94, respectively, whereas in the VATS group, the scores were 2.55 ± 0.82, 2.90 ± 0.64, and 2.45 ± 0.82, respectively. The SITS group had significantly better patient satisfaction scale scores than the VATS group at 24 and 48 hours postoperatively (*p *= 0.045, *p *= 0.041, respectively; Table 3).

## Discussion

The major difficulty with SITS stems from the need for the surgeon to adapt to the new method of instrumentation. The SITS technique is not a naturally ergonomic technique, because the traditional thoracoscopic principles of triangulation are lost. Because both the operating instruments and thoracoscope are introduced through the same incision, and on the same axis, the operator and assistant often impede the movements of each other. This is not helped by current instrumentation, which has not been designed with the single-incision approach in mind. Instruments often interfere with each other, not only within the pleural space, but also extrapleurally, where attachments such as the camera light lead often impede movement. This makes clear and accurate communication between surgeon and assistant essential, especially with regard to intraoperative complications such as bleeding.

In our experience, mild adhesions could be managed with diathermy and reticulated instruments, and good hemostasis is possible with the SITS approach. Bleeding from the cupola or apex of the lung can be treated with diathermy, and bleeding from aberrant vessels between cupola and apex of lung can be managed with application of the EndoCLIP (Covidien, USA) device. If hemostasis is difficult to achieve with the SITS approach, we advocate the insertion of additional thoracoscopic ports to improve surgical dexterity, with conversion to a mini-thoracotomy procedure if necessary.

In the future, we hope these difficulties will be alleviated by the development of new, inline instruments, which will avoid interference. Also, increasing the length of the camera shaft would allow the assistant to stand comfortably with his or her hands away from those of the operating surgeon.

In our report, we have shown SITS for the management of PSP to be a safe and effective technique. To date, the apparent advantages of the SITS technique are primarily related to patient satisfaction. Although three-port thoracoscopic surgery for PSP has been well established, SITS seems to be better choice. Further work in the form of randomized controlled trials are needed to evaluate the potential benefits of this new technique before its use can be widely recommended.

## Conclusion

Although three-port thoracoscopic surgery for PSP is well established, SITS results in better patient satisfaction and decreased postoperative pain in the treatment of PSP.

## Competing interests

PRC, JGC, YSL, HCH, JST, CYC, and HYF have no conflicts of interest or financial ties to disclose. The authors alone are responsible for the content and writing of the paper.

## Authors' contributions

PRC wrote the manuscript and revised it. CKC and YSL collected and analyzed the data, HCH carried out the surgical intervention of patients. JST cared the patients in the study. CYC carried out coordination between authors. HYF established the study structures.
